# Antibiotic use among patients admitted to tertiary hospitals in Uganda: a trend analysis of 2020–2023 point prevalence surveys

**DOI:** 10.1136/bmjopen-2025-110251

**Published:** 2026-03-09

**Authors:** Suzan Nakasendwa, Jonathan Mayito, Vivian Twemanye, Conrad Tumwine, Reuben Kiggundu, Ronald Galiwango, Elly Nuwamanya, James Muleme, Flavia Dhikusooka, Herman Mwanja, Ellon Twinomuhwezi, Harriet Akello, Morries Seru, Hope Mackline, Dathan M Byonanebye, Francis Kakooza, Andrew Kambugu

**Affiliations:** 1 Makerere University Infectious Diseases Institute, Kampala, Uganda; 2 Makerere University School of Public Health, Kampala, Uganda; 3 Republic of Uganda Ministry of Health, Kampala, Uganda

**Keywords:** Antibiotics, Guideline Adherence, Medication Adherence, Inpatients, Public Hospitals, Prescriptions

## Abstract

**Abstract:**

**Objective:**

Limited data exist on temporal changes in antibiotic use in low and middle-income countries. We evaluated trends in antibiotic use at tertiary hospitals in Uganda.

**Design:**

Retrospective trend analysis of a repeated point prevalence survey (PPS).

**Setting and participants:**

This study utilised antibiotic use data from quarterly PPS conducted among inpatients at nine regional referral hospitals in Uganda between October 2020 and December 2023.

**Outcome measures:**

We determined the proportions of antibiotic use, prescriptions guided by culture and sensitivity tests (CST), WHO AWaRe (*Access, Watch and Reserve*) categories, and prescriptions without documented indication. Linear regression was used to derive slope coefficients and 95% confidence interval (CI).

**Results:**

Of 15,154 patients surveyed, 8,892 (58.7%) received systemic antibiotics. The median age was 23 years (IQR: 11–38), 5,338 (60.5%) were female, and 4,583 (51.5%) were on treatment for infectious syndromes, including sepsis (1,400, 15.7%) and pneumonia (867, 9.8%). The drug utilisation index (DU75) consisted of ceftriaxone, metronidazole, gentamicin and ampicillin, which accounted for 76.9% (12,291/15,989) of total antibiotic use. The distribution of prescribed antibiotics was 46.6% Access, 45.5% Watch, 0.1% Reserve and 7.7% unrecommended combinations. Overall, 5,402 (60.8%) prescriptions were aligned with national guidelines, 2,147 (24.1%) prescriptions were issued without an indication, and CST guided 271 (3%) prescriptions. Over time, there was no significant change in antibiotic prescription prevalence (slope=0.09, CI −0.93 to 1.10) and prescriptions without indication (slope=−0.70, CI −1.79 to 3.98). However, adherence to treatment guidelines (slope=2.06, CI 0.14 to 3.98) and prescriptions based on CST results (slope=0.62, CI 0.12 to 1.13) significantly increased, while ‘*Watch’* antibiotics prescriptions decreased (slope=−0.40, CI −0.63 to –0.17).

**Conclusions:**

The antibiotic prescription rate remained high, with no significant change over time. Improvements were seen in adherence to treatment guidelines, use of CST and reduced use of ‘*Watch’* antibiotics. Strengthening antibiotic stewardship is recommended to further improve practices.

**STRENGTHS AND LIMITATIONS OF THIS STUDY**

- Our study provides potentially useful data on temporal changes in antibiotic use in a developing country.
- Our study considered multi-year and multi-centre data from national surveys, providing a comprehensive assessment of antibiotic use in tertiary healthcare facilities using the standardised WHO PPS methodology.
- Demographic and treatment data were not collected for patients not receiving antibiotics on the day of the survey, which limited our ability to interrogate the determinants of antibiotic use among patients.
- The study only includes data for tertiary hospitals and may not be generalisable to the entire health sector.

## Introduction

 Antimicrobial resistance (AMR) is a global public health challenge,^1^ and antibiotic misuse is a major contributor to its emergence, especially in low- and middle-income countries (LMICs).^2^ The inappropriate antibiotic use was 38% in LMICs.^3^ AMR leads to prolonged illness,^4^ increased healthcare costs, higher morbidity and mortality, and it negatively impacts quality of life.^5^ The factors contributing to inappropriate antibiotic use include poor health systems, prescribers’ lack of knowledge and inadequate diagnostic capacity, all of which are prevalent in LMICs.^6^

Antibiotic stewardship programmes are key to optimising antibiotic use and curbing the rise of AMR. These programmes ensure appropriate prescribing, reduce unnecessary use and promote evidence-based treatment.^7^ In Uganda, the implementation of an antimicrobial stewardship programme in six hospitals between June 2019 and July 2022 led to notable reductions in unnecessary antibiotic use for upper respiratory tract infections (URTI) and fewer antibiotics per patient treated for urinary tract infections (UTI).^8^ Despite these promising outcomes, widespread adoption of antimicrobial stewardship programmes faces several challenges. These include a shortage of human resources, inadequate laboratory capacity for microbiological testing, limited governmental funding and a lack of national guidelines.^9^ In a weak antimicrobial development pipeline, minimising inappropriate antibiotic use remains the most practical and immediate strategy to combat AMR, especially in resource-limited settings.^10 11^

Antibiotic use among hospitalised patients in sub-Saharan Africa is widespread, with studies showing high prevalence. A systematic review and meta-analysis study estimated that 64% of inpatients in the region receive antibiotics.^12^ In Uganda, antibiotic-prescribing practices often deviate from national treatment guidelines, with compliance as low as 30% reported among inpatients.^13^ The most commonly used antibiotics among hospitalised patients were ceftriaxone, metronidazole, ciprofloxacin, amoxicillin and azithromycin.^13 14^

Promoting appropriate antibiotic prescribing and dosing is a central goal of both the WHO Global Action Plan (GAP) on AMR^15^ and the Uganda National Action Plan on AMR (NAP-AMR),^16^ which has been in implementation since 2018. Although Uganda has introduced various antimicrobial stewardship interventions to support this goal,^17^ their impact has not been fully evaluated. To address this gap, we analysed surveillance data on antibiotic use from nine regional referral hospitals (RRH) over a 3-year period to assess trends and the potential impact of interventions to optimise antibiotic use.

## Methods

### Study design and setting

This was a retrospective trend analysis of repeated point prevalence surveys (PPS) on antibiotic use collected between October 2020 and December 2023 at nine RRHs in Uganda.^18^ These RRHs were anonymised to A, B, C, D, E, F, G, H and J, and they typically provide clinical care for complex and referral cases. The methods for these surveys have been reported elsewhere.^13^

### Study population

This study was conducted among patients admitted to five major wards: gynaecology, maternity, medical, paediatrics and surgical, and those who were on at least one systemic (oral, intravenous, intramuscular, subcutaneous, inhalation, transdermal, rectal, sublingual, intranasal, intrathecal/epidural or intraosseous) antibiotic at the time of the survey.

### Data collection procedures

Point prevalence surveys (PPS) were conducted every 3 months using the WHO Methodology for PPS in Hospitals, Version 1.1 tool.^19^ During each survey, the medical records of all patients admitted were reviewed to determine eligibility. Data on antibiotic use (ABU) were abstracted from the medical records of all patients receiving systemic antibiotics, admitted before 8:00 am on the day of the survey and not discharged at the time of data collection. Patients admitted after 08:00 a.m. or discharged before the survey were excluded. The study variables included age, gender, ward, dates of admission, number and name of prescribed antibiotics and data on ABU indicators, such as antibiotic use by WHO AWaRe (*Access, Watch, Reserve*) categories, adherence to treatment guidelines, prescriptions guided by culture and sensitivity test results and antibiotic prescription without a documented clinical indication (no reason in the notes).

### Statistical analysis

The data were prepared and analysed using R software V. 4.4.2.^20^ Continuous variables were summarised using the median with IQRs for skewed distributions and using the mean with SD for approximately normally distributed data. Frequencies and percentages were used to describe categorical variables and the indicators. Antibiotic use was presented as proportions against five indicators: (1) prevalence of antibiotic use, (2) antibiotic prescription per WHO AWaRE categories, (3) antibiotic prescriptions adhering to treatment guidelines, (4) prescriptions based on culture and sensitivity test results, and (5) diagnoses that did not warrant treatment with an antibiotic. These descriptives per indicator were disaggregated by Ward and by facility. The indicators were computed as shown in the online supplemental methods.

An antibiotic prescription was considered adherent to treatment guidelines if the antibiotic choice for a given diagnosis was in line with the recommendations of the 2016 Uganda Clinical Guidelines,^21^ which served as the standard of care at the time of the study. The use of culture and sensitivity test results was assessed by determining whether an antibiotic was prescribed based on the test results.

A simple linear regression model was used to derive the slope coefficient and 95% CIs for the trends in (1) adherence to treatment guidelines, (2) antibiotic prescription without indication, (3) rates of prescription based on culture and sensitivity test results and (4) antibiotic use per WHO AWaRe categorisation.

### Patient and public involvement

None.

## Results

A total of 15,154 patients were included in the study, of whom 8,892 (58.7%) received antibiotics. During the analysis, the 15,154 were used as the denominator to derive the prevalence of antibiotic use, while all other antibiotic use parameters were derived using 8,892 as the denominator. The median age (IQR) of patients receiving antibiotics was 23.0 (11.0, 38.0) years, 60.5% (5,338/8,892) of whom were females, 51.5% (4,583/8,892) had an infectious syndrome, 17.6% (1,563/8,892) had urinary catheters, 2.4% (213/8,892) were intubated, and 83.2% (7,396/8,892) had peripheral vascular cannulas (table 1). The most common infectious syndromes were sepsis - 15.7% (1,400/8,892) and pneumonia – 9.8% (867/8,892). Antibiotic prescriptions related to caesarean section were 15.7% (1,400/8,892) (online supplemental table 1).

**Table 1:** Characteristics of patients prescribed at least one antibiotic (n=8892)

Characteristic	Categories	Frequency, n (%)
Age (years)	Median (IQR)	23 (11, 38)
	<1	736 (8.2)
	1–4	998 (11.2)
	5–14	1092 (12.3)
	15–24	1733 (19.5)
	25–34	1546 (17.4)
	35–44	804 (9.0)
	45–54	571 (6.4)
	55–64	400 (4.5)
	65–80	556 (6.3)
	>80	183 (2.1)
	Missing	273 (3.1)
Sex	Male	3486 (39.5)
	Female	5338 (60.5)
	Missing	68 (0.8)
An indwelling device used on patients	Central Vascular Catheter	88 (1.0)
	Urinary Catheter	1563 (17.6)
	Intubation	213 (2.4)
	Peripheral vascular cannular*	7396 (83.2)
Number of antibiotics prescribed per patient	1 antibiotic	3091 (34.8)
	2 antibiotics	4749 (53.4)
	≥3 antibiotics	1052 (11.8)
Number of antibiotic indications (diagnoses)	Median (IQR)	1 (1, 2)
The patient had an infectious syndrome.	Yes	4583 (51.5)
	No	4309 (48.5)
Common Infectious Syndromes	Sepsis	1400 (15.7)
	Pneumonia	867 (9.8)
	Skin and Soft tissue infections	619 (7.0)
	Malaria	575 (6.5)
Duration on antibiotics (days)	Median (IQR)	3 (2, 6)
	<=5	6440 (72.4)
	6–7	940 (10.6)
	8–14	1026 (11.5)
	>14	435 (4.9)
	Missing	51 (0.6)
Timing of the surveys	December 2020	830 (9.3)
	March 2021	646 (7.3)
	June 2021	489 (5.5)
	September 2021	731 (8.2)
	December 2021	719 (8.1)
	March 2022	735 (8.3)
	June 2022	702 (7.9)
	September 2022	796 (9.0)
	December 2022	834 (9.4)
	March 2023	702 (7.9)
	September 2023	824 (9.3)
	December 2023	884 (9.9)

Patients with central vascular catheters, urinary catheters and those with bronchial intubation also had peripheral venous cannulas.

* Did not have any other indwelling device.

Table 2 shows antibiotic prescriptions per WHO AWaRe categorisation and table 3 shows antibiotic use disaggregated by Regional Referral Hospital and ward against key indicators.

**Table 2:** Antibiotic prescriptions per WHO AWaRe categorisation

WHO AWaRe categorisation	Antibiotic	Number prescribed: n=15,989
Access: n=7453	Metronidazole	4,199 (56.3)
	Gentamicin	1,480 (19.9)
	Ampicillin	977 (13.1)
	Amoxicillin	170 (2.3)
	Cloxacillin	153 (2.1)
	Penicillin G	128 (1.7)
	Amoxicillin/Clavulanate	90 (1.2)
	Chloramphenicol	74 (1.0)
	Doxycycline	45 (0.6)
	Nitrofurantoin	44 (0.6)
	Cotrimoxazole	36 (0.5)
	Amikacin	18 (0.2)
	Phenoxymethylpenicillin	12 (0.2)
	Ornidazole	10 (0.1)
	Clindamycin	6 (0.1)
	Cefazolin	5 (0.1)
	Tinidazole	4 (0.1)
	Tetracycline	2 (<0.1)
Watch: n=7274	Ceftriaxone	5,635 (77.5)
	Ciprofloxacin	409 (5.6)
	Cefotaxime	358 (4.9)
	Azithromycin	282 (3.9)
	Piperacillin/Tazobactam	190 (2.6)
	Levofloxacin	174 (2.4)
	Cefixime	92 (1.3)
	Meropenem	41 (0.6)
	Erythromycin	36 (0.5)
	Clarithromycin	19 (0.3)
	Cefuroxime	16 (0.2)
	Imipenem	7 (0.1)
	Vancomycin	7 (0.1)
	Moxifloxacin	4 (0.1)
	Ofloxacin	2 (<0.1)
	Streptomycin	1 (0<1)
	Ceftazidime	1 (<0.1)
Reserve: n=4	Linezolid	4 (100)
Unrecommended: n=1256	Ampicillin/Cloxacillin	853 (67.9)
	Ceftriaxone/Sulbactam	292 (23.3)
	Amoxicillin/Flucloxacillin	98 (7.8)
	Cefoperazone/Sulbactam	13 (1.0)
Not Categorised: n=2	*Nitazoxanide	2 (100)

* Nitazoxanide does not have an AWaRe category.

AWaRe: Access, Watch, and Reserve.

**Table 3:** Antibiotic use disaggregated by Regional Referral Hospital and ward against key indicators

Categories	Number of patients admitted	Number of patients prescribed at least one antibiotic (a)	The number of patients prescribed an antibiotic is consistent with national guidelines	Number of patients prescribed antibiotics without indication	Number of antibiotic prescriptions based on culture and sensitivity test results	Total number of antibiotics prescribed (b)	The average number of antibiotics prescribed (b/a)	Number of patients receiving parenteral antibiotics
Overall, n (%)	15 154	8892 (58.7)	5402 (60.8)	2147 (24.1)	271 (3.0)	15 989	1.8	8233 (92.6)
Regional Referral Hospitals								
A, n (%)	1633	988 (60.5)	496 (50.2)	303 (30.7)	3 (0.3)	1657	1.7	908 (91.9)
B, n (%)	1124	654 (58.2)	420 (64.2)	139 (21.3)	32 (4.9)	1177	1.8	607 (92.8)
C, n (%)	1457	924 (63.4)	663 (71.8)	184 (19.9)	72 (7.8)	1795	1.9	874 (94.6)
D, n (%)	992	602 (60.7)	345 (57.3)	160 (26.6)	22 (3.7)	1083	1.8	540 (89.7)
E, n (%)	1800	1134 (63.0)	748 (66.0)	229 (20.2)	18 (1.6)	2170	1.9	1046 (92.2)
F, n (%)	1546	1049 (67.9)	635 (60.5)	245 (23.4)	17 (1.6)	1801	1.7	935 (89.1)
G, n (%)	1610	924 (57.4)	516 (55.8)	221 (23.9)	63 (6.8)	1706	1.9	861 (93.2)
H, n (%)	2846	1385 (48.7)	721 (52.1)	365 (26.4)	44 (3.2)	2587	1.9	1295 (93.5)
J, n (%)	2146	1232 (57.4)	858 (69.6)	301 (24.4)	0 (0)	2013	1.6	1167 (94.7)
Ward								
Gynaecology, n (%)	1561	941 (60.3)	592 (62.9)	222 (23.6)	37 (3.9)	1815	1.9	830 (88.2)
Maternity, n (%)	2453	1614 (65.8)	1089 (67.5)	285 (17.7)	40 (2.5)	3387	2.1	1539 (95.4)
Medical, n (%)	3223	1776 (55.1)	912 (51.4)	506 (28.5)	64 (3.6)	2828	1.6	1611 (90.7)
Paediatrics, n (%)	3932	2385 (60.7)	1606 (67.3)	570 (23.9)	55 (2.3)	3977	1.7	2324 (97.4)
Surgical, n (%)	3985	2176 (54.6)	1203 (55.3)	564 (25.9)	75 (3.5)	3982	1.8	1929 (88.6)
Indwelling device								
Peripheral Cannula	–	7130 (80.8)	4200 (58.9)	1792 (25.1)	201 (2.8)	–	–	–
Others*	–	1762 (19.8)	1202 (68.2)	355 (20.1)	70 (4.0)	–	–	–

Note: % are row percentages.

* Includes central vascular catheter, urinary catheter and intubation.

### Antibiotics per patient

The median duration of antibiotic treatment was 3.0 (IQR: 2.0, 6.0) days (table 1), while the average number of antibiotics prescribed per patient was 1.8 (15,989/8,892) (table 3). Maternity wards had the highest average prescriptions per patient, 2.1 (3,387/1,614), followed by the gynaecology ward at 1.9 (1,815/941). Ceftriaxone, 35.2% (5,635/15,989), and metronidazole, 26.3% (4,199/15,989), were the most prescribed antibiotics (table 2). Over 90% (92.9%; 8,233/8,892) of patients received parenteral antibiotics, while 19.8% (1,762/8,892) had indwelling devices other than a peripheral cannula (table 3).

### Prescriptions according to the WHO AWaRe Categorisation

The DU75 consisted of ceftriaxone, metronidazole, gentamicin and ampicillin, which together accounted for 76.9% (12,291/15,989) of total antibiotic use. ‘Access’ antibiotics were prescribed in 46.6% (7,453/15,989) of participants, ‘Watch’ antibiotics in 45.5% (7,274/15,989), while only 0.1% (4/15,989) of the prescribed antibiotics were from the ‘Reserve’ category (table 3). The most prescribed antibiotics per WHO AWaRe categorisation was ‘Access’: metronidazole – 56.3% (4,199/7,453) and gentamycin – 19.9% (1,480/7,453); ‘Watch’: ceftriaxone – 77.5% (5,635/7,274) and ciprofloxacin – 5.6% (409/7,274); ‘Reserve’: linezolid – 100% (4/4) and ‘Unrecommended’: ampicillin/cloxacillin – 67.9% (853/1256) and ceftriaxone/sulbactam – 23.3% (292/1,256) (table 2).

### Adherence to treatment guidelines

Adherence of antibiotic prescriptions to the national guidelines was 60.8% (5,402/8,892). There were differences in adherence to guidelines between hospitals and wards, with medical (51.4%, 912/1,776) and surgical wards (55.3%, 1,203/2,176) having the lowest and highest adherence to the national guidelines, respectively (table 3). Overall, 2,147/8,892 (24.1%) patients were prescribed antibiotics for diagnoses that did not warrant their use. Antibiotic prescriptions without indication were highest in medical (28.5%) and surgical wards (25.9%) (table 3).

### Culture and sensitivity test results

Out of 15 989 antibiotics prescribed, 2.2% (352/15,989) were based on culture and sensitivity test results, which varied between hospitals and wards (table 3). The gynaecology ward had the highest proportion of prescriptions (3.9%, 37/941) based on culture and sensitivity test results (table 3).

Regarding the WHO AWaRe categorisation, 2.0% (152/7,453) of ‘Access’ and 2.2% (157/7,274) of ‘Watch’ antibiotic prescriptions were based on culture and sensitivity test results. Of the ‘reserve’ antibiotics, 50% (2/4) were prescribed based on culture and sensitivity test results. The prescription rates deferred across hospitals and wards (table 4).

**Table 4:** Key indicators on antibiotic prescriptions over the evaluation period

Categories	Number of antibiotics prescribed per WHO AWaRe categorisation				Antibiotic prescriptions based on culture and sensitivity test results, per WHO AWaRe categorisation: n=352			
	Access	Watch	Reserve	Unrecommended	Access	Watch	Reserve	Unrecommended
Overall	7453 (46.6)	7274 (45.5)	4 (0.07)	1256 (7.9)	152 (2.0)	157 (2.2)	2 (18.2)	41 (3.3)
Regional referral hospitals								
A	701 (42.3)	866 (52.3)	0	90 (5.4)	1 (0.1)	2 (0.2)	0	0
B	550 (46.7)	539 (45.8)	0	88 (7.5)	14 (2.5)	16 (3.0)	0	2 (2.2)
C	798 (44.5)	884 (49.2)	2 (0.1)	111 (6.2)	51 (6.4)	58 (6.6)	2 (100)	9 (8.1)
D	545 (50.3)	431 (39.8)	1 (0.1)	106 (9.8)	7 (1.3)	8 (1.9)	0	2 (1.9)
E	1085 (50.0)	872 (40.2)	0	213 (9.8)	13 (1.2)	9 (1.0)	0	2 (0.9)
F	840 (46.6)	821 (45.6)	0	140 (7.8)	10 (1.2)	9 (1.1)	0	2 (1.4)
G	703 (41.2)	811 (47.5)	0	191 (11.2)	21 (3.0)	26 (3.2)	0	18 (9.4)
H	1345 (52.0)	1123 (43.4)	1 (0.1)	117 (4.5)	35 (2.6)	29 (2.6)	0	6 (5.1)
J	886 (44.0)	927 (46.1)	0	200 (9.9)	0	0	0	0
Ward								
Gynaecology	875 (48.2)	822 (45.3)	0	118 (6.5)	25 (2.9)	24 (2.9)	0	6 (5.1)
Maternity	1770 (52.3)	1455 (43.0)	0	162 (4.8)	25 (1.4)	17 (1.2)	0	10 (6.2)
Medical	870 (30.8)	1.778 (62.9)	1 (<0.1)	177 (6.3)	26 (3.0)	41 (2.3)	0	7 (3.9)
Paediatrics	2216 (55.7)	1559 (39.2)	0	202 (5.1)	25 (1.1)	34 (2.2)	0	6 (3.0)
Surgical	1722 (43.2)	1660 (41.7)	3 (0.1)	597 (15.0)	51 (3.0)	41 (2.5)	2 (66.7)	12 (2.0)

Note: The percentages were computed per RRH and ward.

AWaRe, Access, Watch and Reserve.

### Trends of antibiotic use

The overall prevalence of antibiotic use did not change over the evaluation period; slope=0.09 (95% CI −0.93 to 1.10). Similarly, the prevalence of antibiotic use across wards did not change significantly over time (online supplemental table 2; figure 2A).

**Figure 1:**
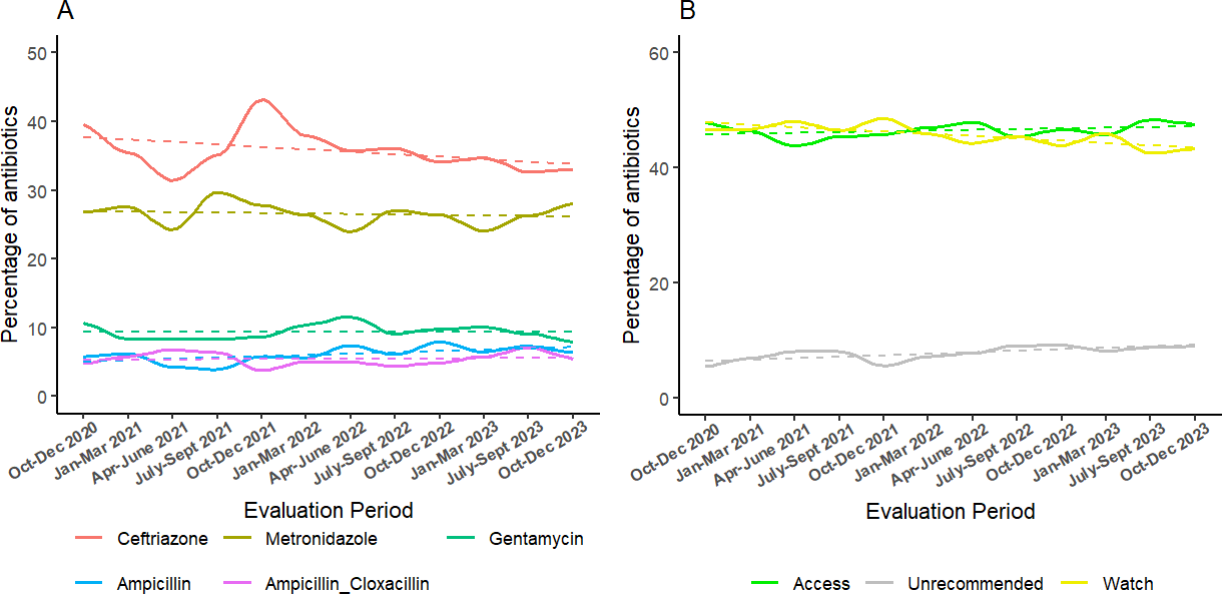
Trends in antibiotic prescription. (A) Percentage of common antibiotic prescriptions; (B) Percentage of prescribed antibiotics per WHO AWaRe categorisation. AWaRe, Access, Watch and Reserve.

Antibiotic prescriptions by WHO AWaRe categories varied over time. ‘Access’ and ‘unrecommended’ antibiotic prescriptions significantly increased; slope=0.13 (95% CI −0.12, 0.37) and slope=0.26 (95% CI 0.08, 0.43), respectively, whereas ‘Watch’ antibiotics significantly declined over the evaluation period; slope=−0.40 (95% CI −0.63 to –0.17) (figure 1B).

**Figure 2:**
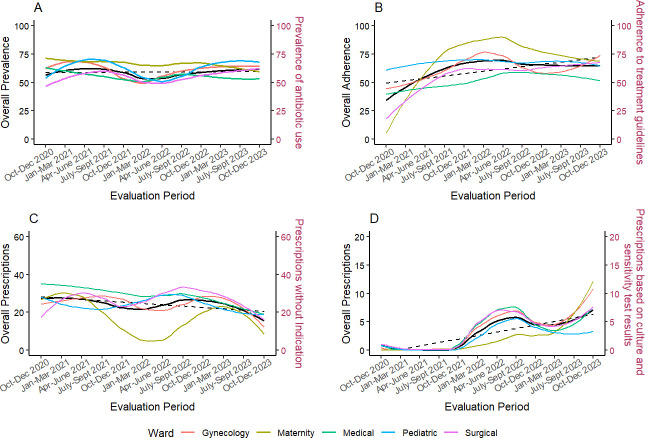
Trends in overall antibiotic prescriptions and per ward. (A) Prevalence of antibiotic prescriptions; (B) Percentage of antibiotic prescriptions adherent to the treatment guidelines; (C) Percentage of patients prescribed an antibiotic without an indication; (D) Percentage of patients prescribed antibiotics based on culture and sensitivity test results. The black solid line represents the overall estimates, the black dotted line is the fitted line from the overall estimates, and the coloured lines represent estimates per ward.

Overall, adherence to treatment guidelines increased significantly over the evaluation period (slope=2.06, CI 0.14 to 3.98). The magnitude of the improvement differed between wards. Statistically significant increases were observed in the medical ward (slope=1.36, CI 0.18, 2.54) and surgical ward (slope=2.45, CI 1.31, 5.59); however, no significant change was observed in the gynaecology, maternity and paediatric wards (figure 2B).

Overall, there was a non-significant reduction in antibiotic prescriptions without indication over the evaluation period (slope=−0.85, CI −2.13 to 0.43). However, a statistically significant decrease was observed in the medical ward (slope=−1.39, CI −2.15 to –0.63), indicating a ward-specific improvement (figure 2C).

Antibiotic prescriptions based on culture and sensitivity test results showed a slight increase over the evaluation period; slope=0.62 (95% CI 0.12 to 1.13), particularly in the gynaecology ward; slope=0.88 (95% CI 0.07 to 1.69), and maternity wards; slope=0.78 (95% CI 0.12 to 1.44) (figure 2D).

## Discussion

This secondary analysis of PPS data aimed to explore the potential impact of antimicrobial stewardship interventions implemented in Uganda’s tertiary hospitals by assessing antibiotic use trends.

Our findings show that more than half of hospitalised patients received almost two antibiotics, with the overall prescription rate remaining high and unchanged over time, and adherence to treatment guidelines and CST-guided prescriptions significantly increased. The observed high and static rate of antibiotic use among inpatients is consistent with findings from similar studies, such as one involving patients in teaching and non-teaching hospitals,^22^ where antibiotic use was similarly prevalent. The trend reflects broader challenges many LMICs face where implementing effective AMS programmes remains difficult due to limited human resources, inadequate laboratory capacity and clinical guidelines that often do not account for the complex case mix in tertiary hospitals.^9^ In Uganda, AMS efforts are largely donor-funded, suggesting a need to strategically target facilities with the highest antibiotic use to maximise impact. Given limited funding, antimicrobial stewardship in the country should target facilities with high antimicrobial use prevalence to achieve a greater impact.^16^

The use of antibiotics in the WHO Watch category remained high but declined over the observation period, suggesting a gradual shift toward greater use of Access antibiotics. On the other hand, the use of the Access category increased over time, but remained below the WHO target of 60%, indicating a shift from Watch to Access antibiotic use. These observed trends might be attributed to the ongoing stewardship programmes in these facilities.^23^ The slow decline in overall antibiotic use and *Watch* antibiotics may stem from continued barriers to diagnostic testing: only 3% of prescriptions were based on microbiological testing. Despite this, the increase in culture and sensitivity testing during the study period is a positive sign. Strengthening laboratory capacity, including the availability of equipment, supply chains, infrastructure and skilled personnel, will be critical for the continued success of AMS programmes.^24^ These findings also point to broader systemic changes. The improvement in culture-guided prescribing may reflect both shifts in prescriber behaviour and enhanced operational capacity, such as improved supply chains for laboratory reagents^25^ and investments in laboratory infrastructure and human resources. These factors likely reinforce each other, contributing to the observed trends.

In this study, we observed that adherence to treatment guidelines increased significantly over the evaluation period, while prescriptions without indication declined, particularly in the medical and surgical wards. These positive changes may result from the implementation of regular prescription audits and clinician feedback and suggest that interventions targeting clinician prescription behaviour or antibiotic supply are complementary to efforts to strengthen rational antibiotic use.^26 27^ The above trends indicate a positive shift in clinical practice, with healthcare workers increasingly following established guidelines and using diagnostic testing to guide treatment decisions.^28^

While our study provides potentially useful data on temporal change in antibiotic use in a developing country, it has several limitations.

The first limitation concerns the data-collection methodology within each hospital. Although all participating facilities conducted their point prevalence surveys during the same predefined quarterly periods to ensure temporal comparability, data were collected ward-by-ward (one ward per day), meaning the full hospital survey was completed over 5 days. This multi-day approach, while logistically necessary in large regional referral hospitals and aligned with established protocols, deviates from an ideal single-day “point” prevalence measure. Day-of-week variations in admission volumes, patient case-mix (eg, higher proportions of new admissions early in the week), and prescribing practices (eg, delayed antibiotic reviews or de-escalation on weekends due to reduced staffing) could introduce minor temporal biases. Such variability might have a subtle influence on estimates of antibiotic prevalence, distribution of WHO AWaRe categories, guideline adherence, documentation of indications, or utilisation of culture and sensitivity testing across survey rounds.

Second, the study includes only data from tertiary hospitals and may not reflect the entire health sector. Despite the limitations above, our study considered multi-year and multi-centre data from national surveys, providing a comprehensive assessment of antibiotic use in tertiary healthcare facilities using the standardised WHO PPS methodology. This strengthens the reliability of our findings and supports broader efforts to institutionalise antimicrobial use surveillance at the national and regional levels.

## Conclusion

In Uganda’s tertiary hospitals, antibiotic use remained high and unchanged over time. However, there were significant improvements in adherence to treatment guidelines, CST-guided prescriptions and prescriptions of ‘*Watch’* antibiotics, likely due to ongoing stewardship interventions. There is, however, a need to further strengthen antibiotic stewardship interventions and increase access to microbiology testing to promote evidence-based prescribing, enhance adherence to treatment guidelines and expand access to diagnostic tools.

## Supplementary material

10.1136/bmjopen-2025-110251 online supplemental file 1

## Data Availability

Data are available upon reasonable request.
