# Supplementary material for: Antibiotic use among patients admitted to tertiary hospitals in Uganda: a trend analysis of 2020–2023 point prevalence surveys
Source: BMJ Open. 2026 Mar 9;16(3):e110251. doi: 10.1136/bmjopen-2025-110251 (PMC12983728; doi:10.1136/bmjopen-2025-110251)
Supplement: online supplemental file 1 [file bmjopen-16-3-s001.docx]

**Supplemental material**

**Supplementary methods: Computation of key indicators**

1. **Overall prevalence of Antibiotic use**

$$\boldsymbol{Prevalence}=\frac{Number of patients at ward with atleast one antibiotic prescribed}{Total number of patients at the ward} X 100$$

2. **Antibiotics use by WHO AWaRe (*Access, Watch, Reserve*) category**

$$\boldsymbol{Percentage}=\frac{Number of antibiotics prescribed under each AWaRe class}{Overall total number of antibiotics prescribed} X 100$$

3. **Adherence to treatment guidelines**

$$\boldsymbol{Percentage}=\frac{Number of patients prescribed antibiotics adherent to treatment guidelines}{Total number of patients on antibiotic} X 100$$

4. **Use of culture and sensitivity test results per WHO AWaRe categorization.**

$$\boldsymbol{Percentage}=\frac{Total antibiotics prescribed based on culture and sensitvity test}{Total number of antibiotics prescribed per AWaRe category} X 100$$

5. Antibiotic prescription without indication

$$\boldsymbol{Percentage}=\frac{Total patients with Antibiotic prescriptions without indication}{Total number of patients on antibiotics} X 100$$

**Supplemental Table 1: Characterizing Patient Diagnoses**

| **Diagnosis** | **Frequency (%)** |
| --- | --- |
| Sepsis* | 1400 (15.7) |
| Maternal or Fetal Diagnoses for Caesarean Section | 1366 (15.4) |
| Pneumonia* | 867 (9.8) |
| Skin and Soft Tissue Infections* | 619 (7.0) |
| Malaria* | 575 (6.5) |
| Post Traumatic Injuries | 559 (6.3) |
| Sexually Transmitted Diseases* | 451 (5.1) |
| Urinary tract Infections* | 405 (4.6) |
| Anemia | 336 (3.8) |
| Abscess* | 287 (3.2) |
| Sickle Cell Disease | 263 (3.0) |
| Upper Respiratory Tract Infections* | 252 (2.8) |
| Early gestational complications for surgery | 221 (2.5) |
| Intestinal Obstruction | 218 (2.5) |
| Liver Disease | 202 (2.3) |
| Central Nervous System Infections | 200 (2.3) |
| Gastritis | 196 (2.2) |
| Surgical Site Infections* | 189 (2.1) |
| Cancer | 163 (1.8) |
| Peritonitis* | 161 (1.8) |
| Gastrointestinal Infections* | 158 (1.8) |
| Diarrhoea | 133 (1.5) |
| Malnutrition | 124 (1.4) |
| Diabetes Melitus | 111 (1.2) |
| Kidney Disease | 97 (1.1) |
| Heart faliure | 94 (1.1) |
| Bone/Joint Infections* | 88 (1.0) |
| HIV | 73 (0.8) |
| Hypertension | 63 (0.7) |
| Tuberculosis | 62 (0.7) |
| Stroke (Cardiovascular Accident) | 55 (0.6) |
| Cardiovascular Infections | 27 (0.3) |
| Chronic Obstructive Pulmonary Disease (COPD) | 21 (0.2) |
| Asthma | 16 (0.2) |

** Infectious syndrome*

**Supplemental Table 2: Trend estimates in antibiotic use stratified by ward**

| **Categories** | | **Overall** | **Ward** | | | | |
| --- | --- | --- | --- | --- | --- | --- | --- |
|  |  |  | **Gynecology** | **Maternity** | **Medical** | **Pediatrics** | **Surgical** |
|  |  | **Slope (95% Confidence Interval)** | | | | | |
| **Prevalence of antibiotic use** | | 0.09 (-0.93, 1.10) | -0.18 (-2.15, 1.80) | -0.83 (-1.72, 0.06) | -0.67 (-1.81, 0.46) | 0.43 (-1.34, 2.19) | 0.74 (-0.63, 2.10) |
| **% of adherence to treatment guidelines** | | 2.06 (0.14, 3.98) * | 1.71 (-0.27, 3.69) | 3.93 (-1.00, 8.86) | 1.36 (0.18, 2.54) * | 0.25 (-0.96, 1.46) | 2.45 (1.31, 5.59) * |
| **% of patients with antibiotic prescription without indication** | | -0.70 (-1.79, 0.39) | -0.58 (-1.78, 0.64) | -0.17 (-3.94, 1.60) | -1.33 (-1.99, -0.68) | -0.48 (-1.92, 0.97) | -0.11 (-1.91, 1.68) |
| **% of patients’ prescriptions based on culture and sensitivity test results** | | 0.62 (0.12, 1.13) * | 0.88 (0.07, 1.69) * | 0.78 (0.12, 1.44) * | 0.64 (-0.18,1.46) | 0.36 (-0.08, 0.83) | 0.64 (-0.04, 1.32) |
| **Commonly prescribed Antibiotics** | **Ceftriaxone** | -0.36 (-0.94, 0.20) | -0.21 (-0.87, 0.50) | -0.49 (-1.30, 0.21) | -0.07 (-2.21, 0.06) | 0.08 (-0.76, 0.83) | -0.28 (-0.96, 0.37) |
|  | **Metronidazole** | -0.07 (-0.41, 0.65) | 0.17 (-0.36, 0.49) | -0.53 (-0.88, 0.01) | 0.27 (-0.28, 0.30) | 0.04 (-0.23, 0.77) | -0.03 (-0.56, 0.91) |
|  | **Gentamycin** | -0.01 (-0.23, 0.95) | 0.12 (-0.27, 0.49) | 0.08 (-0.27, 0.62) | -0.05 (-0.23, 0.55) | -0.07 (-0.58, 0.75) | -0.05 (0.70, 0.01) |
|  | **Ampicillin** | 0.01 (0.005, 0.04) * | 0.30 (0.07, 0.02) * | 0.63 (0.18, 0.01) | -0.09 (-0.32, 0.39) | 0.17 (-0.54, 0.60) | -0.02 (-0.15, 0.71) |
|  | **Ampicillin/Cloxacillin** | 0.02 (-0.17, 0.79) | -0.20 (-0.47, 0.13) | 0.05 (-0.21, 0.67) | -0.04 (-0.29, 0.76) | 0.19 (-0.06, 0.13) | -0.04 (-0.47, 0.82) |
| **% of antibiotics prescribed per WHO AWaRe categorization** | **Access** | 0.13 (-0.12, 0.37) | 0.46 (0.19, 0.74) * | 0.24 (-0.20, 0.69) | 0.10 (-0.69, 0.84) | 0.14 (-0.72, 1.01) | -0.03 (-0.42, 0.36) |
|  | **Watch** | -0.40 (-0.63, -0.17) * | -0.60 (-1.01, -0.19) * | -0.66 (-1.16, -0.15) * | -0.40 (-0.93, 0.13) | -0.34 (-1.23, 0.56) | -0.23 (-0.67, 0.22) |
|  | **Not recommended** | 0.26 (0.08, 0.43) * | 0.46 (0.19, 0.74) * | 0.24 (-0.20, 0.69) | 0.08 (-0.69, 0.84) | 0.14 (-0.72, 1.01) | -0.03 (-0.42, 0.36) |

**** Statistically significant at P<0.05,* % = Percentage**
