# Supplementary material for: Antibiotic use among patients admitted to tertiary hospitals in Uganda: a trend analysis of 2020–2023 point prevalence surveys
Source: BMJ Open. 2026 Mar 9;16(3):e110251. doi: 10.1136/bmjopen-2025-110251 (PMC12983728; doi:10.1136/bmjopen-2025-110251)
Supplement: Supplementary file 2 [file bmjopen-2025-110251.reviewer_comments.pdf]

PEER REVIEW HISTORY

BMJ Open publishes all reviews undertaken for accepted manuscripts. Reviewers are asked to complete a checklist review form and are provided with free text boxes to elaborate on their assessment. These free text comments are reproduced below.

ARTICLE DETAILS

Title (Provisional)

Antibiotic use among patients admitted to tertiary hospitals in Uganda: a trend analysis of 2020–2023 point prevalence surveys

Authors

Nakasendwa, Suzan; Mayito, Jonathan; Twemanye, Vivian; Tumwine, Conrad; Kiggundu, Reuben; Galiwango, Ronald; Nuwamanya, Elly; Muleme, James; Dhikusooka, Flavia; Mwanja, Herman; Twinomuhwezi, Ellon; Akello, Harriet; Seru, Morries; Mackline, Hope; Byonanebye, Dathan M; Kakooza , Francis; Kambugu, Andrew

---

VERSION 1 - REVIEW

**Reviewer**	**1**
**Name**	**Mapunjo, Siana**
**Affiliation**	**Ministry of Health, Community Development, Gender, Elderly and Children, Dodoma, Tanzania.**
**Date**	**27-Oct-2025**
**COI**	**None**

---

1. Abstract

The abstract lacks critical data in the results section. It should summarize the key numerical findings, including total number of patients surveyed, percentage receiving antibiotics, most common indications, and most frequently prescribed antibiotic classes.

Recommendation:

Revise the abstract to include essential quantitative results such as:

• Total number of patients surveyed and percentage receiving antibiotics.

• Indications (HAI, CAI, surgical prophylaxis etc.).

• Commonest diagnosis (e.g., sepsis, pneumonia).

• Most frequently used antibiotic(s) (e.g., metronidazole, ceftriaxone).

• AWaRe category proportions (to include Not Recommended antibiotic).

2. Methodology – WHO PPS Clarification

• There is inconsistency regarding the study population. According to WHO PPS methodology, all patients present in the ward at 08:00 on the day of the survey should be included, whether or not they are on antibiotics.

• However, your paper indicates inclusion of only patients who were receiving at least one systemic antibiotic, creating a denominator problem.

Recommendation:

Clarify the study population. If only patients on antibiotics were included, then 8,892 cannot be 58.7%; 58.7% implies that this number is part of a larger total. Specify the total number of patients in the wards at 08:00 (the true denominator).

Also, clarify the meaning of "data were abstracted from the prescription without indication." If this means that prescriptions lacked documented clinical indications, rephrase for clarity.

3. Primary and Secondary Outcomes

• You indicated "prescriptions without documented indication" as a key outcome, but did not present corresponding data in the results.

Recommendation:

Include findings on the percentage or number of prescriptions lacking a recorded indication.

4. Missing WHO PPS Indicators

• WHO PPS requires reporting of the reason for antibiotic prescription e.g., surgical prophylaxis, medical prophylaxis, community-acquired infection, healthcare-associated infection, or no indication.

Recommendation:

Add a table or summary paragraph showing the distribution of antibiotic use by indication type. This data is critical for understanding prescribing patterns and stewardship opportunities.

5. AWaRe Classification

• The reported AWaRe distribution (Access 47.8%, Watch 44.4%, Reserve 0.1%) totals 92.3%. The missing 7.7% should be clarified.

Recommendation:

• Explain what constitutes the remaining 7.7% (likely “Unclassified” or “Not Recommended” antibiotics).

• Use WHO’s current terminology — refer to “Not Recommended” antibiotics instead of “Unclassified.”

• Suggest including a policy implication statement on phasing out “Not Recommended” antibiotics from the national Essential Medicines List (EML). E.g. Ampicilin/cloxacillin

6. Key Infectious Syndromes and DU75/DU90

• Important findings like sepsis (15.7%) and pneumonia (9.8%) are hidden in the results section. These should be highlighted in the abstract as major drivers of antibiotic use.

• Only one antibiotic (metronidazole) was highlighted, as the most used antibiotic. while WHO recommends presenting the DU75% or DU90% (top antibiotics accounting for 75–90% of total use).

Recommendation:

Include in the abstract and results a summary of the most common infectious syndromes and the top-used antibiotics (DU90% or DU75%).

7. Discussion – Population Inclusion

• You note as a limitation that data were not collected for patients not receiving antibiotics. However, as per WHO PPS protocol, all patients in the ward at 08:00 must be included.

Recommendation:

Acknowledge this as a deviation from WHO PPS methodology and justify the reason (e.g., resource limitation, modified protocol). This should be clearly stated as a methodological limitation rather than a data limitation.

8. Use of Outdated National Guidelines

• The study references the 2016 Ugandan Standard Treatment Guidelines, which are outdated.

Recommendation:

Acknowledge this as a limitation and discuss how using an older guideline might have affected antibiotic classification or appropriateness assessment. Consider referencing that other countries (e.g., Tanzania, 2021) have updated versions, highlighting the need for Uganda to update its own.

**Reviewer**	**2**
**Name**	**Sunderland, Bruce**
**Affiliation**	**Curtin University, School of Pharmacy**
**Date**	**27-Nov-2025**
**COI**	**None**

This paper provides useful information on antibiotic prescribing in major hospitals in Uganda

The study is a retrospective repeated cross-sectional study, which should be indicated in the Abstract.

It is unclear whether the same groups collected the data, then subsequently analysed it. Collection and management of raw data should require ethics approval, although a waiver may be provided for individual patient consent. If ethical approval was provided to collect the data and de-identified data were provided for analysis then ethics approval is not required. This should be clarified.

The Introduction provides an adequate introduction for the study.

In the Methodology it is noted the 2016 Clinical Guidelines were used for data collected from 2020 to 2023. Ugandan guidelines appear to be regularly updated. Why were guidelines that were current for the year each data set were collected not used?

It is noted the guidelines provided the antibiotic of choice, dosage and often duration. When stating as adherent to guidelines, providing data on each element assists with antibiotic stewardship programs.

Was surgical prophylaxis included in the data set? It is noted there were few prescriptions for cefazolin which is often a suitable antibiotic for this purpose.

It is noted the highest prescribed antibiotic was ceftriaxone which is one that should be limited where possible, as resistance is evident already in some countries.

The consistency of prescribing across the nine hospitals is notable.

Was the data collected for each hospital done in one day, or over several days? Were the same days always used for the survey? Some days may have more new admissions affecting prescribing. This may affect the limitations.

VERSION 1 - AUTHOR RESPONSE

Reviewer: 1

Dr. Siana Mapunjo, Ministry of Health, Community Development, Gender, Elderly and Children, Dodoma, Tanzania. Comments to the Author:

1. Abstract

The abstract lacks critical data in the results section. It should summarize the key numerical findings, including total number of patients surveyed, percentage receiving antibiotics, most common indications, and most frequently prescribed antibiotic classes.

Recommendation:

Revise the abstract to include essential quantitative results such as:

• Total number of patients surveyed and percentage receiving antibiotics.

Addressed in the abstract *(line 23)*

• Indications (HAI, CAI, surgical prophylaxis etc.)

The analysis and presentation of the data focused on rational use by condition and not by category of conditions. Presenting the CAI, HAI proportions over time may not add new information.

• Commonest diagnosis (e.g., sepsis, pneumonia).

These were included *(line 25 to 26)*

• Most frequently used antibiotic(s) (e.g., metronidazole, ceftriaxone).

These were included *(line 26 to 27)*

• AWaRe category proportions (to include Not Recommended antibiotic).

The number and percentage of 'Not recommended antibiotics' has been added *(line 27)*

2. Methodology – WHO PPS Clarification

• There is inconsistency regarding the study population. According to WHO PPS methodology, all patients present in the ward at 08:00 on the day of the survey should be included, whether or not they are on antibiotics.

This study is looking at trends of antibiotic use; it is a secondary analysis of surveillance data from PPS specifically among patients on antibiotics. *(line 95 to 104)*

• However, your paper indicates inclusion of only patients who were receiving at least one systemic antibiotic, creating a denominator problem.

This study is looking at trends of antibiotic use; it is a secondary analysis of surveillance data from PPS specifically among patients on antibiotics. *(line 102 to 104)*

Recommendation:
Clarify the study population. If only patients on antibiotics were included, then 8,892 cannot be 58.7%; 58.7% implies that this number is part of a larger total. Specify the total number of patients in the wards at 08:00 (the true denominator).
The first line of the results presents the prevalence of antibiotic use as 58.7% i.e. (8,892 on antibiotics out of 15154 patients admitted). The subsequent analyses of adherence to treatment guidelines, WHO AWaRe, culture and sensitivity were referring to the patients on antibiotics and the denominator was 8,892. *(line 134 to 135)*

Also, clarify the meaning of "data were abstracted from the prescription without indication." If this means that prescriptions lacked documented clinical indications, rephrase for clarity.
The statement has been edited to provide this clarity. *(line 100 to 102)*

3. Primary and Secondary Outcomes

• You indicated "prescriptions without documented indication" as a key outcome but did not present corresponding data in the results.
Recommendation:
Include findings on the percentage or number of prescriptions lacking a recorded indication.
Results of this indicator are available in Table 3 and presented under the section on adherence to treatment guidelines. (*line 169 to 172)*

4. Missing WHO PPS Indicators

• WHO PPS requires reporting of the reason for antibiotic prescription e.g., surgical prophylaxis, medical prophylaxis, community-acquired infection, healthcare-associated infection, or no indication.
Recommendation:
Add a table or summary paragraph showing the distribution of antibiotic use by indication type. This data is critical for understanding prescribing patterns and stewardship opportunities.
While the PPS methodology recommends this, the analysis and presentation of the data focused on rational use (right patient, right drug, right dose, etc) by condition and not by category of conditions. Presenting the CAI, HAI proportions over time does not add new information to the objective of this manuscript.

5. AWaRe Classification

• The reported AWaRe distribution (Access 47.8%, Watch 44.4%, Reserve 0.1%) totals 92.3%. The missing 7.7% should be clarified.
Recommendation:
• Explain what constitutes the remaining 7.7% (likely "Unclassified" or "Not Recommended" antibiotics).
These are described and included in Table 2.

• Use WHO's current terminology — refer to "Not Recommended" antibiotics instead of "Unclassified."
This has been adjusted in Table 2 and elsewhere.

• Suggest including a policy implication statement on phasing out "Not Recommended" antibiotics from the national Essential Medicines List (EML). E.g. Ampicilin/cloxacillin.
The text has been edited to reflect the standard nomenclature i.e. unrecommended fixed-dose combinations. *(Table 2)*

6. Key Infectious Syndromes and DU75/DU90

• Important findings like sepsis (15.7%) and pneumonia (9.8%) are hidden in the results section. These should be highlighted in the abstract as major drivers of antibiotic use.
These have been updated in the abstract and in the results section. *(line 25 to 26)*

• Only one antibiotic (metronidazole) was highlighted as the most used antibiotic. while WHO recommends presenting the DU75% or DU90% (top antibiotics accounting for 75–90% of total use).

Recommendation:
Include in the abstract and results a summary of the most common infectious syndromes and the top-used antibiotics (DU90% or DU75%).

These have been updated in the abstract and results. The DU75 segment comprised the top four antibiotics, which collectively accounted for 77% of total antibiotic use. *(line 26 to 28)*

7. Discussion – Population Inclusion

• You note as a limitation that data were not collected for patients not receiving antibiotics. However, as per WHO PPS protocol, all patients in the ward at 08:00 must be included.

Recommendation:
Acknowledge this as a deviation from WHO PPS methodology and justify the reason (e.g., resource limitation, modified protocol). This should be clearly stated as a methodological limitation rather than a data limitation.

Thank you for this comment. We acknowledge that this is not a limitation since this analysis only focused on patients using antibiotics. The statement regarding patients included in this analysis has been edited to ensure clarity *(line 252 to 263)*

8. Use of Outdated National Guidelines

• The study references the 2016 Ugandan Standard Treatment Guidelines, which are outdated.

Recommendation:
Acknowledge this as a limitation and discuss how using an older guideline might have affected antibiotic classification or appropriateness assessment. Consider referencing those other

countries (e.g., Tanzania, 2021) have updated versions, highlighting the need for Uganda to update its own.

At the time of data collection, the 2016 Uganda Clinical Guidelines were the current guidelines. The 2023 UCG were released in 2024.

Reviewer: 2

Dr. Bruce Sunderland, Curtin University

Comments to the Author:

This paper provides useful information on antibiotic prescribing in major hospitals in Uganda.

The study is a retrospective repeated cross-sectional study, which should be indicated in the Abstract

This has been adjusted in the abstract and body of the manuscript.*(line 15 and 89)*

It is unclear whether the same groups collected the data, then subsequently analysed it. Collection and management of raw data should require ethics approval, although a waiver may be provided for individual patient consent. If ethical approval was provided to collect the data and de-identified data were provided for analysis then ethics approval is not required. This should be clarified.

This has been clarified. This study was approved by the Infectious Diseases Institute Research Ethics Committee (IDI-REC-2023-67) for the conduct of research using secondary data from Point Prevalence Surveys. *(line 280 to 283)*

The Introduction provides an adequate introduction for the study.
Noted with thanks

In the Methodology it is noted the 2016 Clinical Guidelines were used for data collected from 2020 to 2023. Ugandan guidelines appear to be regularly updated. Why were guidelines that were current for the year each data set were collected not used?

At the time of data collection, the 2016 Uganda Clinical Guidelines were the standard of care. The 2023 UCG were released in 2024.

It is noted the guidelines provided the antibiotic of choice, dosage and often duration. When stating as adherent to guidelines, providing data on each element assists with antibiotic stewardship programs.

In the PPS data collection tool, there is a single variable on adherence to the clinical guideline, which is recorded as a YES/NO. Prescriptions were considered adherent to treatment guidelines (YES) if the choice of antibiotic was in line with the guidelines.

Was surgical prophylaxis included in the data set? It is noted there were few prescriptions for cefazolin which is often a suitable antibiotic for this purpose.

Although not indication categories were not included in the analysis, surgical prophylaxis was one of the indications for antibiotic use. In this data set, only five prescriptions were for Cefazolin and not exclusively for surgical prophylaxis.

It is noted the highest prescribed antibiotic was Ceftriaxone, which is one that should be limited where possible, as resistance is evident already in some countries.

This is a correct observation. With low diagnostic capacity, and limited human resource, empiric treatment with broad-spectrum antibiotics that are administered once a day such as Ceftriaxone is common. *(Table 2)*

The consistency of prescribing across the nine hospitals is notable.

Was the data collected for each hospital done in one day, or over several days? Were the same days always used for the survey? Some days may have more new admissions affecting prescribing. This may affect the limitations.

In each survey round, all participating facilities conducted their PPS during the same week to ensure comparability across time points. Within each hospital, data were collected ward by ward, with one ward surveyed per day, meaning the full hospital PPS was completed over five days, typically Monday to Friday. The same wards were included in every round for each hospital. We acknowledge the subtle effects of day-of-the-week variations and have included this in the limitations. *(line 258 to 263)*

VERSION 2 - REVIEW

Reviewer **1**

Name **Mapunjo, Siana**

Affiliation **Ministry of Health, Community Development, Gender, Elderly and Children, Dodoma, Tanzania.**

Date **19-Jan-2026**

COI

---

1. Methodology (Data collection procedures) 9

During each survey, medical records of all patients admitted were reviewed.

As per the WHO Point Prevalence Survey (PPS) methodology, medical records are reviewed only for eligible inpatients. Eligible patients are defined as those admitted to the ward before 08:00 a.m. on the day of the survey and not discharged at the time of data collection. Patients admitted after 08:00 a.m. or discharged before the survey is excluded. So you need to review this section.

Data on antibiotic use (ABU) were abstracted from the medical records of all patients admitted before 8:00 am on the day of the survey and receiving systemic antibiotic treatment.

Please, review this data collection procedure as per WHO PPS protocol All eligible inpatients, defined as patients admitted to the ward before 08:00 a.m. on the day of the survey and not discharged at the time of data collection, were included. The 08:00 a.m. cut-off is applied strictly as an eligibility criterion for patient & antibiotic inclusion, in accordance with the WHO PPS methodology.

2. Results

The study included 15,154 patients, of these, 8,892 (58.7%) received antibiotics.

As a limitation and in other section you have mentioned that demographic and treatment data were not collected for patients not receiving antibiotics on the day of the survey. But in this result section we have a numerator (patient prescribed antibiotic) and denominator (total surveyed patients),

Contradiction: You report total patients surveyed, yet say no data were collected for patients not on antibiotics.

Correct interpretation

- Denominator: all eligible patients present at 08:00 a.m.
- Numerator: patients receiving ≥1 systemic antibiotic

Recommendation: The methodology section should be revised to accurately describe the study procedures and ensure consistency with the reported results.

Piperacillin/Tazobactam and nitazoxanide AWaRe classification

Piperacillin/tazobactam was classified as an Access antibiotic in the manuscript; however, according to the WHO AWaRe classification, it belongs to the Watch group. This misclassification should be corrected, and the AWaRe-based analyses should be repeated accordingly.

In addition, nitazoxanide is not included in the WHO AWaRe classification and therefore should not be listed under "not recommended" antibiotics. A comprehensive review of the antibiotic list is recommended to ensure that all agents are correctly classified according to the WHO AWaRe framework.

.

Penicillin V

The manuscript should consistently use WHO-recommended generic names. "Penicillin V" does not appear in the WHO AWaRe list; instead, **phenoxymethylpenicillin** is listed. Please confirm and revise accordingly.

3. Limitation

Demographic and treatment data were not collected for patients not receiving antibiotics on the day of the survey, which limited our ability to interrogate the determinants of antibiotic use among the patients.

According to the WHO Point Prevalence Survey (PPS) protocol, demographic and admission-related data are collected for all eligible patients, including age, sex, and admission history. For all patients not receiving antibiotics, the remaining sections of the questionnaire, such as indication for antibiotic use and the antibiotic treatment form are not completed. This section should therefore be reviewed to ensure consistency with the WHO PPS protocol. Otherwise in the methodology section you may state that you used a modified WHO PPS protocol.

**Reviewer**	**2**
**Name**	**Sunderland, Bruce**
**Affiliation**	**Curtin University, School of Pharmacy**
**Date**	**07-Jan-2026**
**COI**	

Most of the suggestions made have been addressed.

With respect to guideline adherence, I consider it should be clearly stated this only related to antibiotic selection and not dosage or duration.

There are minor areas where the English language could be tidied up, especially the capitalising of some antibiotic names.

VERSION 2 - AUTHOR RESPONSE

Reviewer 1:

With respect to guideline adherence, I consider it should be clearly stated this only related to antibiotic selection and not dosage or duration.

This was rectified. (lines 122 to 124)

There are minor areas where the English language could be tidied up, especially the capitalising of some antibiotic names.

The entire manuscript has been revised.

Reviewer 2:

1. **Methodology (Data collection procedures)**

During each survey, medical records of all patients admitted were reviewed.

As per the WHO Point Prevalence Survey (PPS) methodology, medical records are reviewed

only for eligible inpatients. Eligible patients are defined as those admitted to the ward before 08:00 a.m. on the day of the survey and not discharged at the time of data collection. Patients admitted after 08:00 a.m. or discharged before the survey are excluded. So you need to review this section.

This has been revised. (Lines 100 to 110)

Data on antibiotic use (ABU) were abstracted from the medical records of all patients admitted before 8:00 am on the day of the survey and receiving systemic antibiotic treatment. Please, review this data collection procedure as per WHO PPS protocol All eligible inpatients, defined as patients admitted to the ward before 08:00 a.m. on the day of the survey and not discharged at the time of data collection, were included. The 08:00 a.m. cut-off is applied strictly as an eligibility criterion for patient & antibiotic inclusion, in accordance with the WHO PPS methodology.

This has been revised. (Lines 100 to 110)

2. **Results**

The study included 15,154 patients, of these, 8,892 (58.7%) received antibiotics. As a limitation and in other section you have mentioned that demographic and treatment data were not collected for patients not receiving antibiotics on the day of the survey. But in this result section we have a numerator (patient prescribed antibiotic) and denominator (total surveyed patients),

Contradiction: You report total patients surveyed, yet say no data were collected for patients not on antibiotics.

Correct interpretation: Denominator: all eligible patients present at 08:00 a.m. Numerator: patients receiving ≥1 systemic antibiotic

Recommendation: The methodology section should be revised to accurately describe the study procedures and ensure consistency with the reported results.

This has been revised. (Lines 136 to 139)

Piperacillin/Tazobactam and nitazoxanide AWaRe classification

Piperacillin/tazobactam was classified as an Access antibiotic in the manuscript; however, according to the WHO AWaRe classification, it belongs to the Watch group. This misclassification should be corrected, and the AWaRe-based analyses should be repeated accordingly.

In addition, nitazoxanide is not included in the WHO AWaRe classification and therefore should not be listed under "not recommended" antibiotics. A comprehensive review of the antibiotic list is recommended to ensure that all agents are correctly classified according to the WHO AWaRe framework.

Thank you for identifying this error. The WHO AWaRe classification has been thoroughly revised in Table 4, Figure 1, Supplemental Table 2, and the corresponding interpretation throughout the manuscript.

Penicillin V

The manuscript should consistently use WHO-recommended generic names. "Penicillin V" does not appear in the WHO AWaRe list; instead, **phenoxymethylpenicillin** is listed. Please confirm and revise accordingly.
Penicillin V has been renamed to phenoxymethylpenicillin

3. **Limitation**

Demographic and treatment data were not collected for patients not receiving antibiotics on the day of the survey, which limited our ability to interrogate the determinants of antibiotic use among the patients. According to the WHO Point Prevalence Survey (PPS) protocol, demographic and admission-related data are collected for all eligible patients, including age, sex, and admission history. For all patients not receiving antibiotics, the remaining sections of the questionnaire, such as indication for antibiotic use and the antibiotic treatment form are not completed. This section should therefore be reviewed to ensure consistency with the WHO PPS protocol. Otherwise in the methodology section you may state that you used a modified WHO PPS protocol.
We do not have this statement in our current version of the manuscript. This was revised in the first version.

VERSION 3 - REVIEW

Reviewer 1

Name Mapunjo, Siana

Affiliation Ministry of Health, Community Development, Gender, Elderly and Children, Dodoma, Tanzania.

Date 29-Jan-2026

COI

Minor comment on Lines 143–144: A total of 15,154 patients were admitted to the wards during the evaluation period, of whom 8,892 (58.7%) received antibiotics.

As per the WHO Point Prevalence Survey (PPS) protocol, not all admitted patients are eligible for inclusion in a PPS study. I recommend revising the sentence to read: "A total of 15,154 patients were included in the study, of whom 8,892 (58.7%) received antibiotics."

Reviewer 2

Name Sunderland, Bruce

Affiliation Curtin University, School of Pharmacy

Date **11-Feb-2026**

COI

The matter raised have been addressed by the authors. However, the text in the Results section Line 143 should be amended to 7396/8892 to give 83.2%.

VERSION 3 - AUTHOR RESPONSE

Reviewer: 1

Minor comment on Lines 143–144: A total of 15,154 patients were admitted to the wards during the evaluation period, of whom 8,892 (58.7%) received antibiotics.

As per the WHO Point Prevalence Survey (PPS) protocol, not all admitted patients are eligible for inclusion in a PPS study. I recommend revising the sentence to read: “A total of 15,154 patients were included in the study, of whom 8,892 (58.7%) received antibiotics.”

This has been revised in line 136

Reviewer: 2

The matter raised have been addressed by the authors. However, the text in the Results section Line 143 should be amended to 7396/8892 to give 83.2%.

This has been revised in line 142
